# Tunable direct band gap photoluminescent organic semiconducting nanoparticles from lignite

**DOI:** 10.1038/s41598-017-18338-2

**Published:** 2017-12-21

**Authors:** Manoj B, Ashlin M Raj, George Thomas Chirayil

**Affiliations:** Department of Physics, Christ University, Bengaluru, Karnataka 560029 India

## Abstract

Fluorescent organic semiconducting dots (OSDs) with tunable particle size and surface functionality are synthesized from lignite by chemical oxidation method followed by ultra-sonication techniques and dialysis. The defects and oxygen functionalities play a vital role in the photoluminescent property of the synthesized nanoparticles along with quantum confinement effect. These nanomaterials are suitable for imaging and chemical sensing applications as there is no photobleaching and quenching even after a continuous UV exposure of 24 hours and storage of 2 years. The excellent excitation dependent luminescence of the synthesized carbon dots can be utilized for making a low-cost carbon-based sensor for Cu^2+^ metal ions sensing. The OSDs show good ratiometric fluorescent sensing and can be used as a reliable probe for the detection of Cu^2+^ ions. They exhibit excellent detection limit of copper ion in acidic solution to a very low concentration of 0.0089 nM. The fluorescent nanodots synthesized from such an abundant and cost-effective precursor exhibiting high copper ion sensitivity is being reported for the first time.

## Introduction

Investigation of the unique optical properties of carbon & graphene-based nanodots has attracted tremendous research interest in recent days. They exhibit fascinating properties like stable fluorescence (FL), chemiluminescence (CL) and electrochemiluminescence (ECL) owing to their size and edge effect^1–4^. Low toxicity, high colloidal stability and low photo quenching make carbon-based organic nanoparticles superior to their inorganic counterpart. Fluorescent carbon dots have immense utility in numerous fields like bio-imaging, fluorescence probing, labeling and energy conversion in photovoltaic^5–15^. Nanocarbon structure has been fabricated from various carbon-based materials including fullerene, glucose, graphite or graphene oxides, carbon nanotubes and carbon fiber by synthesis methods like hydrothermal or electrochemical routes. Even though these are facile approaches, the precursors used are relatively costlier^1–5^. Control of size, structure and optical properties of the carbon dots by tuning the sp^2^ domains in the precursor matrix or carbonaceous product in the preparation process is yet another challenge. Large-scale production of the carbon dots in an optimal way is possible if one could select precursors with preformed sp^2^ domains in it.

Studies have been reported on the synthesis of carbon-based dots from coal and humic substances which are rich in nanocarbon domain. These carbon-based materials are composed of organic compounds which are formed during the coalification or humification process^3–5^. Coal is being considered as an abundantly available precursor for the synthesis of nanotubes, nanoballs, carbon nanodots, onion-like fullerene and graphene quantum dots (GQDs). The size and shape of the carbon crystallites in coal vary with the coalification and the production yield of the nanoparticles is inversely proportional to the coal rank^6–8^. Some of the recent research works have reported the production of novel carbon materials like quantum dots and Nanodiamond from anthracite and bituminous coal^6–8^. Among coals, lignite is one of the low rank coals which is least explored. Lignite is a low-quality energy source owing to its low energy content and inherent mineral matter compared to other types of coal. Being a low-grade coal, it has abundant sp^2^ carbon crystallites embedded in its intrinsic 3D matrix which can be harvested and converted to novel carbon materials in a systematic method. Review of the literature reveals that the GQDs and carbon dots synthesized from lignite form a stable suspension with high colloidal stability^7^. Majority of the studies report the utilization of only the supernatant part of the sample and the residue part is discarded^3,4,9^. Whether these residuals can be converted to carbon dots with unique properties is worth investigating owing to the impact on the cost of production.

It is found that all nano carbon fractions from lignite, the Supernatant, the Residue (after centrifugation) and the Permeate (after dialysis) are oxygenated hexagonal and spherical nanoparticles. They exhibit unique optical properties which are comparable with properties of carbon and graphene dots derived from any synthetic route^8–12^. The synthesized organic dots have very good Cu^2+^ ion sensitivity as low as 0.0089 nM, which make them an ideal label for metal ion detection. The uniqueness of this fluorescent probe is that these organic semiconducting dots (OSDs) are synthesized from low-grade lignite feedstock, which is typically economic compared to other carbon precursors like graphite and high-rank coal. They exhibit ratiometric fluorescence sensing up to the picomolar range which is by far the first study to be reported from lignite or coal-derived carbon dots.

## Results and Discussion

**Figure 1:**
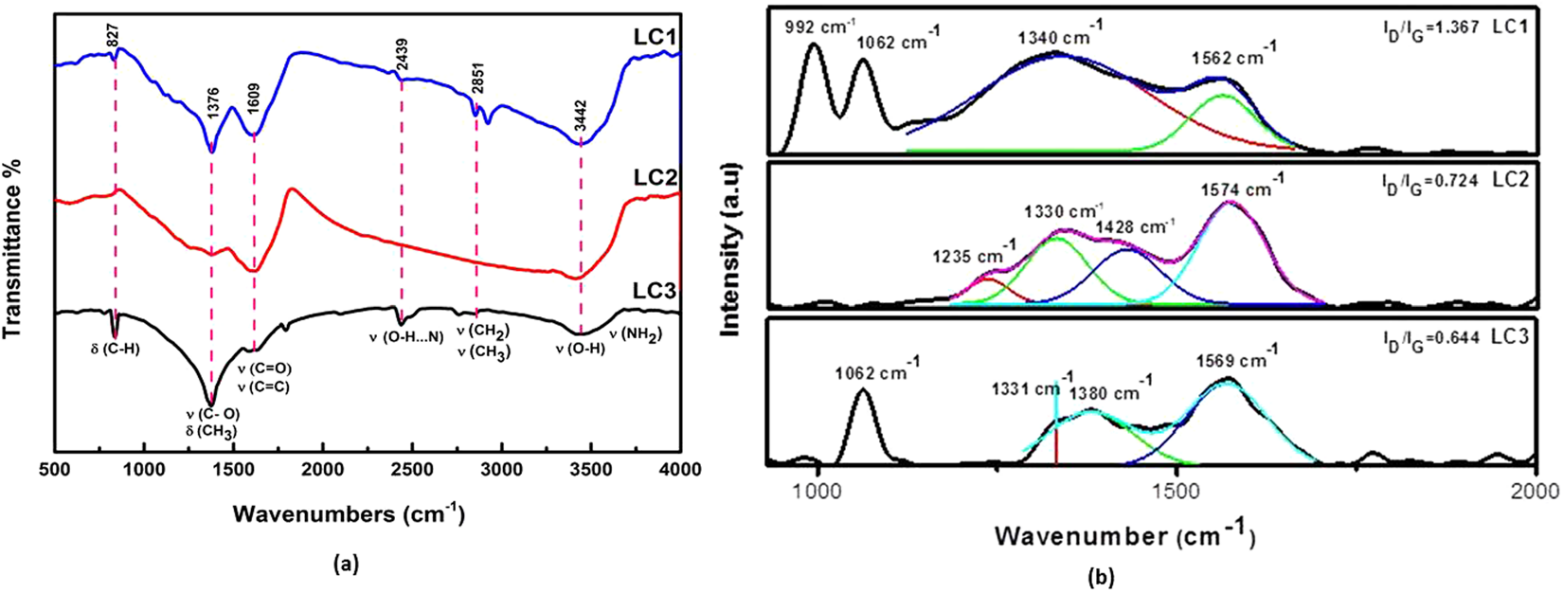
(**a**) FTIR spectra of carbon nanoparticles. (**b**) Raman spectra of LC1, LC2 & LC3 fraction.

The preformed novel carbon nanostructures are extracted by refluxing & ultra-sonication of lignite powder with nitric acid followed by centrifugation, dialysis and heating. The obtained fractions are designated as LC1 (Residue), LC2 (Supernatant) and LC3 (Permeate) (Refer Materials and Methods). Elemental analysis of the samples reveals that they are hydrocarbon with carbon (C), oxygen (O), nitrogen (N) and hydrogen (H) as major elements and smaller quantities of sulfur (S) as a minor element as in any organic matter (see Supplementary Table S1). It is noticed that LC3 has the highest oxygen and nitrogen content, implying that it contains carbonaceous functional groups in abundance. FTIR analysis of the nanostructure is presented in Fig. 1a. The spectra of LC1 and LC2 exhibit similar broad characteristics. The spectra have prominent peaks at 1609 cm^−1^, 1376 cm^−1^, 2851 cm^−1^, 2922 cm^−1^ and 3442 cm^−1^. The band at 1600–1620 cm^−1^ is originated from the conjugated C=C stretching vibration & carbonyl groups present in the nanocarbon. The 1376 cm^−1^ peak is due to the presence of cyclic carbon groups while 1340 cm^−1^ arises due to the C-N (amine) stretching peak. The nitrogen groups are probably originated from lignite or during HNO_3_ oxidation, which acts as a doping agent of nitrogen into organic dots. The band at 2922 cm^−1^ arises from the asymmetric vibration of sp^3^ bond in CH_2_ and CH_3_ groups in the hydrocarbon network^13,14^. The peak at 2851 cm^−1^ is ascribed to the overlapping of symmetric vibration of a sp^3^ band of CH_3_ and CH_2_ functional groups. The 3442 cm^−1^ peak arises from the presence of OH group of alcohols, carboxylic groups and absorbed water. The FT-IR analysis confirms that the synthesized product contains abundant graphitic structure with hydroxyl and carbonyl functional groups^12^. The FTIR analysis result is in support of the result of elemental analysis (see Supplementary Table S1). These nanostructures are highly soluble and form a stable suspension in water due to the high content of carboxyl functional groups.

Raman spectra of all the three samples (LC1, LC2 and LC3) shows strong graphitic peak at 1562 cm^−1^, 1574 cm^−1^ and 1569 cm^−1^ and a defect peak at 1340 cm^−1^, 1330 cm^−1^ and 1380 cm^−1^ respectively (Fig. 1b)^15–20^. These peaks are attributed to the first order scattering of the E_2g_ vibration of graphene and structural defects (or sp^3^ carbon) respectively. The peaks at 1062 cm^−1^ in LC1 & LC3 and 1235 cm^−1^ &1428 cm^−1^ in LC2 are due to trans-polyacetylene (trans-PA) which is an alternate chain of sp^2^ carbon atoms, with a single hydrogen bonded to each carbon^20^. It is inferred that the synthesized nanostructure has defect induced sp^2^ chains with oxygen functional groups attached to it. The intensity ratio I_D_/I_G_ of the three samples are found to be 1.36 (LC1), 0.72 (LC2) and 0.64 (LC3) indicating a lesser degree of defects. (More evidence about defect structure is discussed in the following section-in photoluminescence).

**Figure 2:**
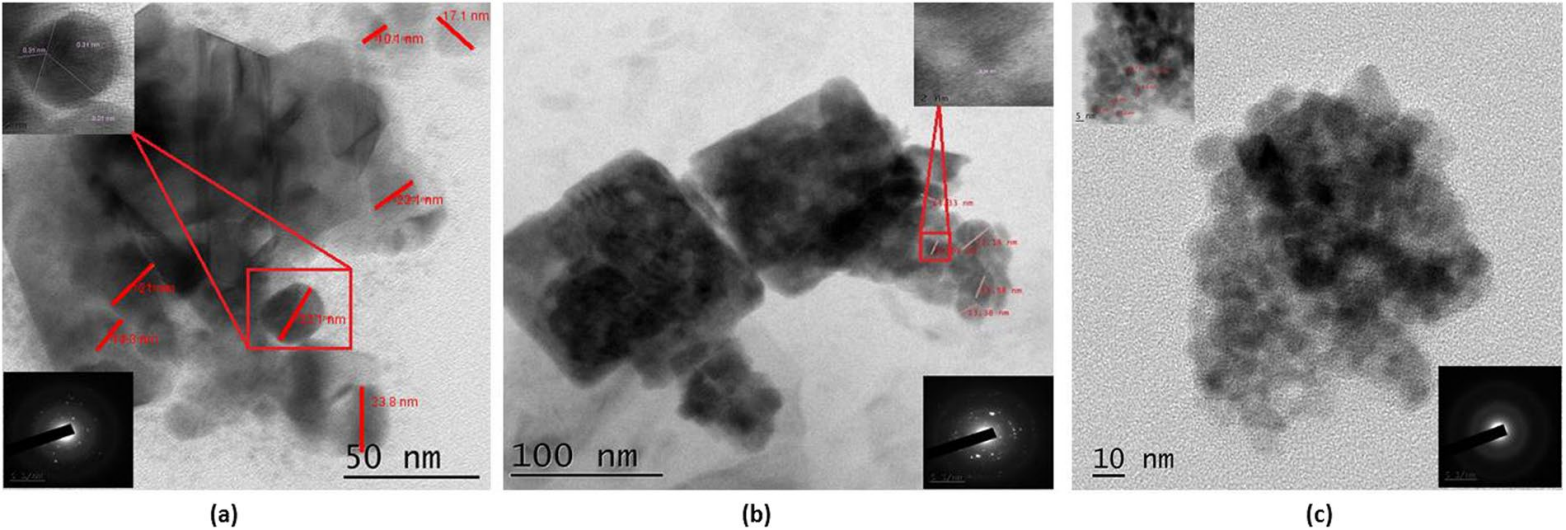
TEM images of (**a**) LC1, (**b**) LC2 and (**c**) LC3. The inset shows TEM image showing lattice fringe and SAED pattern of the marked area. (Scale bar-5 1/nm).

The morphology of the nanostructures is further analyzed by transmission electron microscope (TEM) and is presented in Fig. 2(a–c). One could observe the formation of heterogeneous structures of the shapes hexagonal, pentagonal, trigonal and spherical nanoparticles in the residue (LC1) with the lateral size of the order of 10–23 nm with the majority of particles in the range of 21 nm (Figs 2a and S1). The selected area electron diffraction (SAED) pattern show rings corresponding to the (111) and (200) planes of graphite (see inset). The lattice fringe is measured to be ~0.31 nm and ~0.21 nm (Fig. 2 a inset), confirming the presence of crystalline planes of graphene^3,10–12^.

The TEM analysis of the synthesized nanostructure after dialysis, using the membrane filter (LC2) is presented in the Fig. 2b (Refer Supplementary Information Fig. S2(a–c)). The lateral dimension of the particles is ranging from 10–17 nm with the majority of particles in the size range of 17 nm. The nanoparticles are stacked to form nanosheets of cubic structure. The lattice fringe is measured to be ~0.21 nm and ~0.24 nm. The corresponding SAED pattern show rings which can be attributed to the polycrystalline nature of nanocarbon. The TEM image of LC3 is depicted in Fig. 2c (Supplementary information Fig. S3(a,b)). One could see a large collection of spherical nanoparticles with a size of the order of 5 nm. The particles are homogeneous and have uniform size with a tendency to agglomerate. The lattice fringe is measured to be ~0.24 nm. The TEM analysis shows that nanostructure in LC3 is a mixture of amorphous and nanocrystalline organic dots while that in LC1 and LC2 are nano-crystalline in nature.

**Figure 3:**
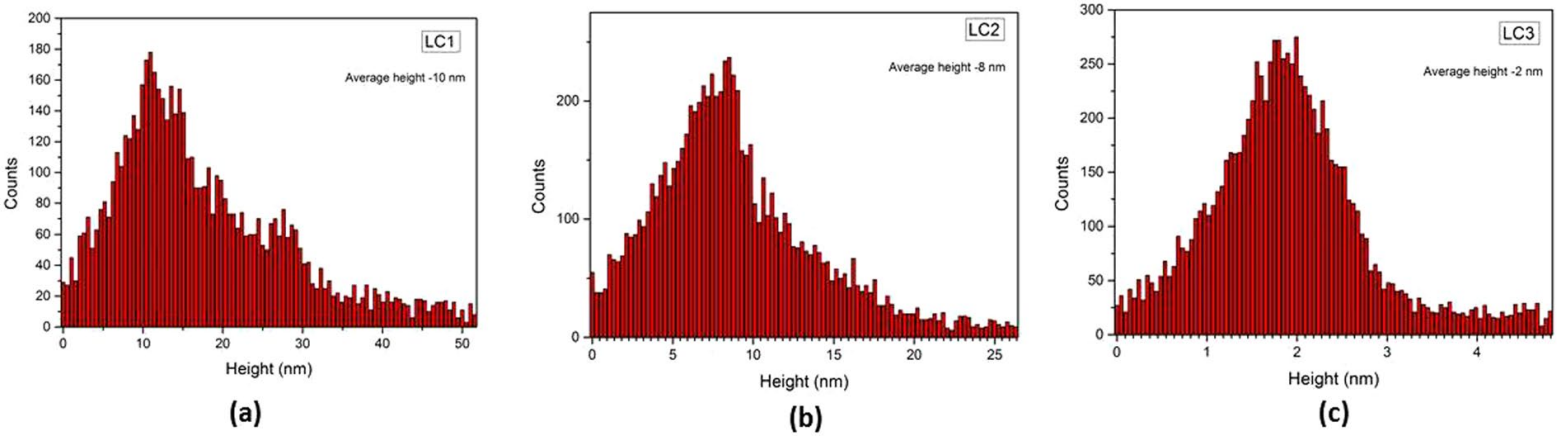
Height profile analysis of nanodots from lignite by AFM analysis.

The height profile of the synthesized carbon nanoparticles is determined by atomic force microscopy (AFM) and is presented in Fig. 3(a–c). The carbon nanostructure in LC1 has a height of the order of 5–10 nm and LC2 has a stacking of about 4–8 nm, attributed to multilayer formation of the nanoparticles. The sample LC3 has about 1–2 nm height owing to the formation of a few layer of carbon.

The CHNS, FTIR, TEM, Raman and AFM analysis confirms the formation of organic nanoparticles with oxygen functionalities attached to the carbon core. The optical properties of this oxygenated carbon nanoparticles are investigated in detail and are presented.

### Optical Properties of the organic nanoparticles

**Figure 4:**
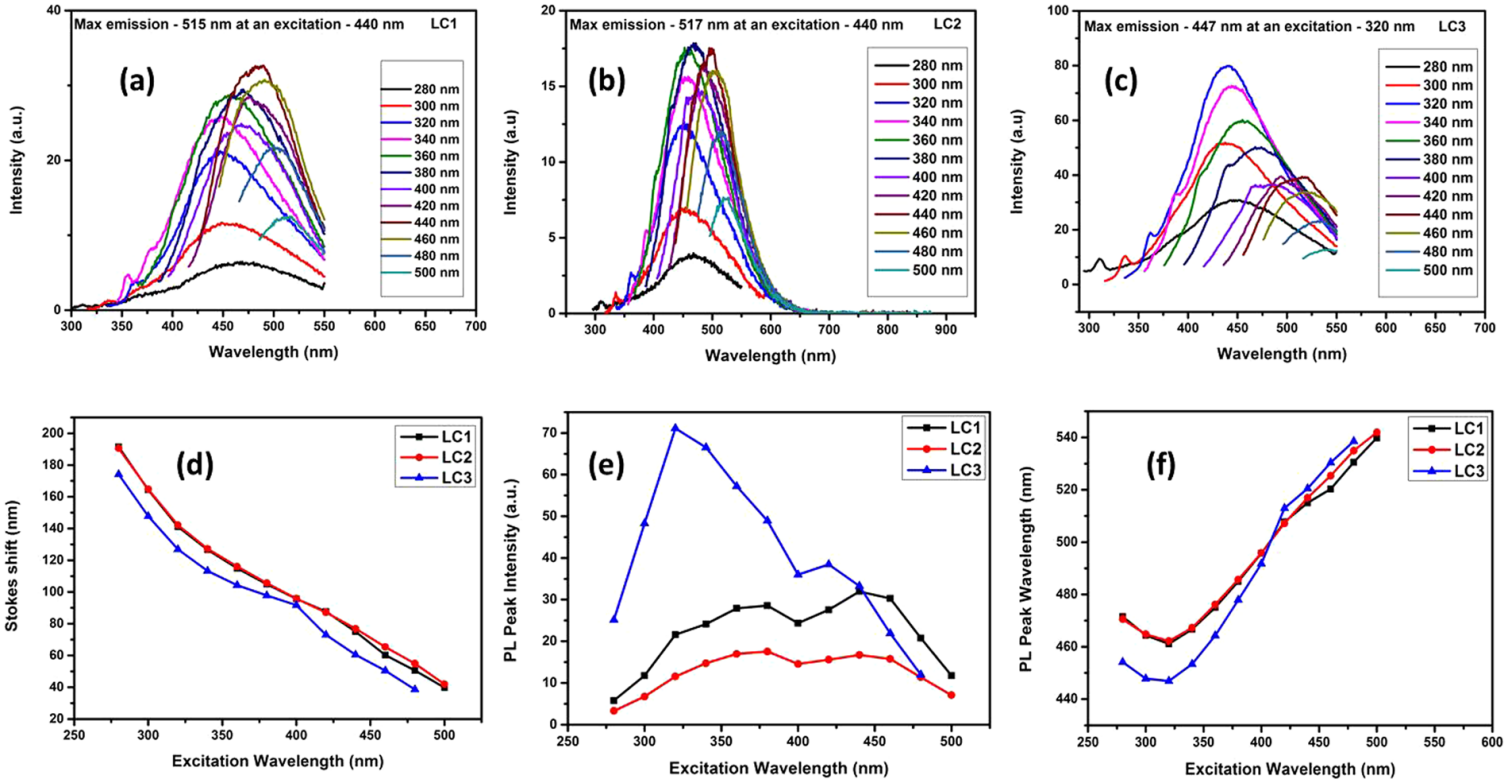
Optical properties of OSDs. PL emission of (**a**) LC1 (**b**) LC2 (**c**) LC3 under different excitation wavelengths from 280 to 500 nm. (**d**) The Stokes shift versus excitation wavelength. (**e**) PL peak intensity versus excitation wavelength. (**f**) PL peak wavelength versus excitation wavelength.

The optical properties of the synthesized organic dots in lignite are investigated by the UV-Visible analysis (see Supplementary Fig. S4(a–c) and photoluminescence (PL) analysis (Fig. 4(a–c)). The photograph of the fluorescence of the nanodots under UV-light exposure is presented in Fig. S5(a,b) (Supplementary Information). A highly green fluorescence is observed in contrast to the pale yellow colored sample under normal visible light. They form a highly stable suspension and possess good photostability even after two years. The stable fluorescence even after long period suggests that they do not photo-bleach and has the potential as a stable imaging probe.

The UV-Visible absorption spectra of the nanodots show a broadening of the UV peak from 220–600 nm. Graphene oxide (GO) sheets normally show a characteristic π-π* transition of the C=C bond and an n-π* transition of the C=O bond at around 230 nm and 270–300 nm respectively. While a typical graphene has only a single π-π* of the C=C bond at 270 nm^21–25^. In the present study two absorption peaks are noticed: one at 232 nm, owing to the π-π* transition of aromatic sp^2^ domains (due to the transition in C=C bond) and a shoulder peak around 280 nm attributed to the n-π* transition of the C=O radical. The band gap of the synthesized nanostructures was determined by plotting the Tauc plot^26^. It is found that the synthesized carbon materials have a band gap of 3.4 eV, 3.2 eV and 3.6 eV for LC1, LC2 and LC3 samples respectively (see Supplementary Fig. S6(a–c)). Thus it is concluded from the Tauc plot that these organic dots are direct band semiconductors in nanoscale size. The energy gap of the nanostructure is found to change with the decrease of average particle size. The spectral analysis (UV-Vis, IR & Raman studies) along with TEM measurement and elemental analysis (CHNS) establish that the lignite-based oxygenated carbon nanostructure is dots with well-defined energy gap (Organic Semiconducting Dots -OSDs). The excitation dependent fluorescence behavior of the OSDs is presented in (Fig. 4(a–c)).

The fluorescence spectra of the OSDs are recorded by varying the excitation wavelength from 280 to 500 nm with an interval of 20 nm. The evolution of its emission with the excitation wavelength can be separated into two parts. For LC2, below ~360 nm, the emission peaks keep their position mainly excitation independent after which they show redshift with increase of excitation wavelength. This trend is noticed in LC1 till ~340 nm and for LC3 it is about ~300 nm. Xiaoming Li *et al*.^21^ attributed this to two leading states - energy gap and surface state transitions. When the excitation energy is higher than the energy gap (lower wavelength), emission from the energy gap transition plays a leading role. When the excitation energy is lower than the energy gap (higher wavelength), surface state emission dominates and contribute to the excitation-dependent behavior. LC3 nanostructure (average particle size 4–5 nm) shows maximum emission at 447 nm (Blue emission). Change in PL intensity, PL peak wavelength and Stokes shift for all the three nanocarbon structures are presented in the Fig. 4(d–f). The maximum intensity of the fluorescence emission was found to be in the green region for LC1 and LC2 while it is in blue region for the LC3. A bathochromic shift to the yellow region is observed with the increase in excitation wavelength which is a special feature of carbon nanodots. It is also observed that with increasing excitation wavelength, the Stokes shift approaches linearly to zero confirming quantum confinement effect in the OSDs^26–32^. It is also suggested that the redshift is attributed to the presence of multiple chromophore/fluorophore system with aromatic and oxidation groups.

**Figure 5:**
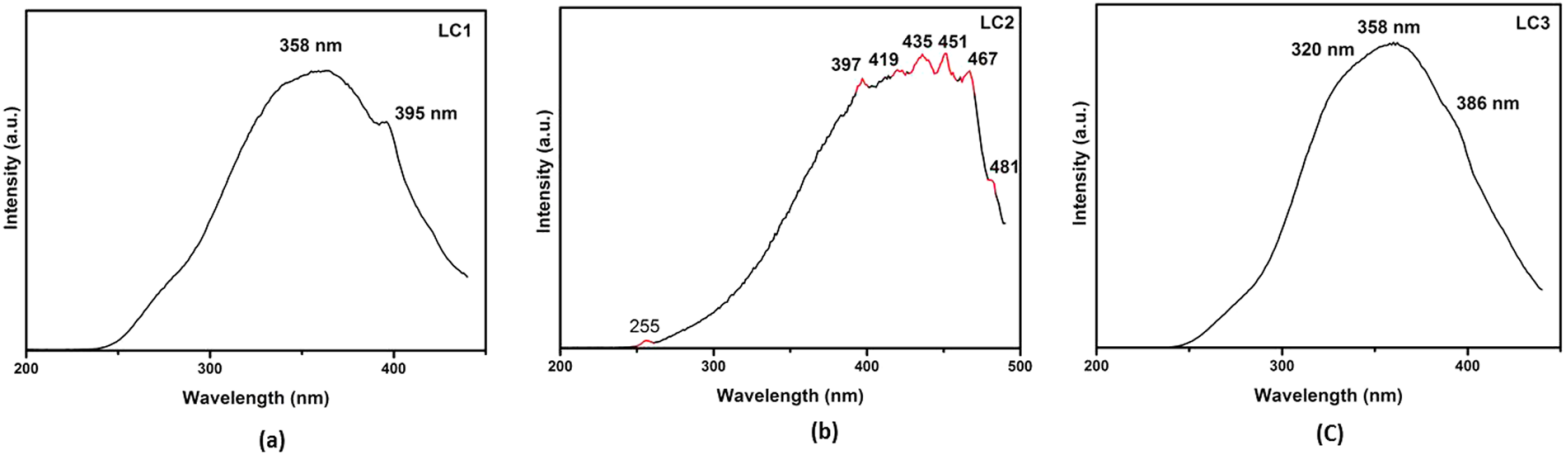
PLE spectrum of the synthesized organic dots (OSDs).

The photoluminescence excitation (PLE) spectrum of the three nanomaterials synthesized is recorded at the respective wavelength of the emission maxima (Fig. 5(a–c)). The PLE spectra of LC1 show two peaks at 358 (3.47 eV) and 395 nm (3.15 eV). The LC2 sample shows a totally different peak at 397 nm (3.13 eV) along with a 467 nm peak (2.66 eV). These two peaks of LC3 are blue shifted to 320 nm and 386 nm (3.89 eV and 3.22 eV). Peng *et al*.^31^, attributed this to the transition from the σ and π-orbital (highest occupied molecular orbitals, HOMOs) to the lowest unoccupied molecular orbital (LUMO).

The carbine ground state multiplicity is responsible for the energy difference (δE) between σ and π-orbital. For a triple state, this energy difference is less than 1.5 eV as reported by other research groups^31,32^. In the present study, the δEs for oxygenated carbon nanoparticles (LC1, LC2 and LC3) with green and blue emissions are 0.32, 0.47 and 0.67 eV respectively. This energy difference is within the required limit for triple carbenes transition. The longer wavelength gap between the two maximum values, provide better sensitivity and makes them ideal for imaging/sensing application.

The luminescence decay profiles of the three synthesized products (LC1, LC2 and LC3) are recorded at room temperature by a time-correlated single photon counting technique (Depicted in Supplementary Table S2). The observed lifetime of LC1 are τ_1_~3.54 ns, τ_2_~0.80 ns and τ_3_~10.28 ns, whereas for LC3, the lifetimes are τ_1_~3.65 ns, τ_2_~0.80 ns and τ_3_~10.14 ns respectively. For LC2, the lifetimes are respectively τ_1_~3.08 ns, τ_2_~0.80 ns and τ_3_~10.28 ns.

The fast emission component (τ_1_) shows the variation in all the three samples. Wang *et al*.^17^ in their recent work, reported that the fast emission component (τ_1_) is due to the oxygen functional groups which might contribute to the PL through electron transition n-σ* (for C-O-C and –OH) and n-π* (for C=O and –COOH). The contribution of the τ_**1**_ component is mainly originates from the high content of oxygen. The value of τ_**2**_ is similar in all the samples and is attributed to the π-π* transition of the aromatic core (sp^2^ skeleton) of the nanodots. The τ_**3**_ emission is found to be consistent and is due to the disordered structure in the sample^31,32^. The observed lifetime of the organic dots in nanoseconds suggest that the synthesized carbon dots are suitable for optoelectronic and biological applications. The fluorescence is mainly due to the combination of oxygen functional groups, defect states and quantum size effect. The emission peak is always subjected to the excitation wavelength which results from the wide distribution of differently sized dots and different emissive traps. The excitation dependent PL makes them an ideal candidate for multi-color imaging and sensing. The fluorescence analysis is in agreement with the findings of Raman analysis. The defect state in the LC1 sample is high compared to LC2 and LC3 due to the non-uniform particle size and shape.

### Variation of Fluorescence with pH

**Figure 6:**
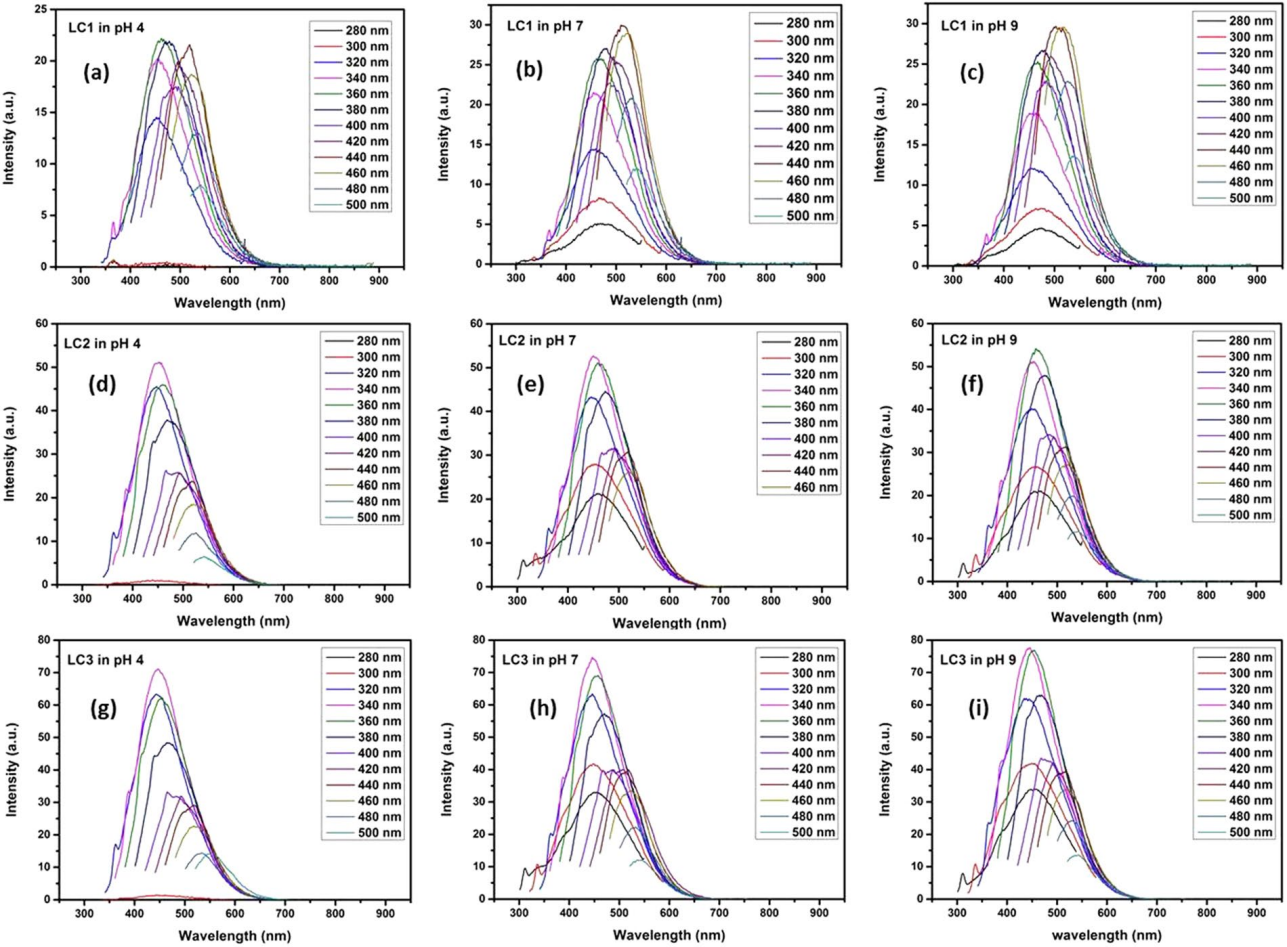
The fluorescence property of OSDs under various pH (**a**–**c**) LC1, (**d**–**f**) LC2 and (**g**–**i**) LC3.

Effect of solvent pH on fluorescence property of the synthesized nanoparticle is investigated at different pH (Acidic-4, Neutral-7 and Basic-9) and the findings are presented in Fig. 6(a–i). It is observed that at neutral and basic pH, the position of the spectra remains unaltered. But at acidic pH (pH-4), there was a shift in the position of the fluorescence spectra. The result is in agreement with the pH dependence fluorescence behavior of coal-derived GQDs reported by Ye *et al*.^2^ in high ranked coals.

### Metal ion sensing capacity of lignite derived OSDs

**Figure 7:**
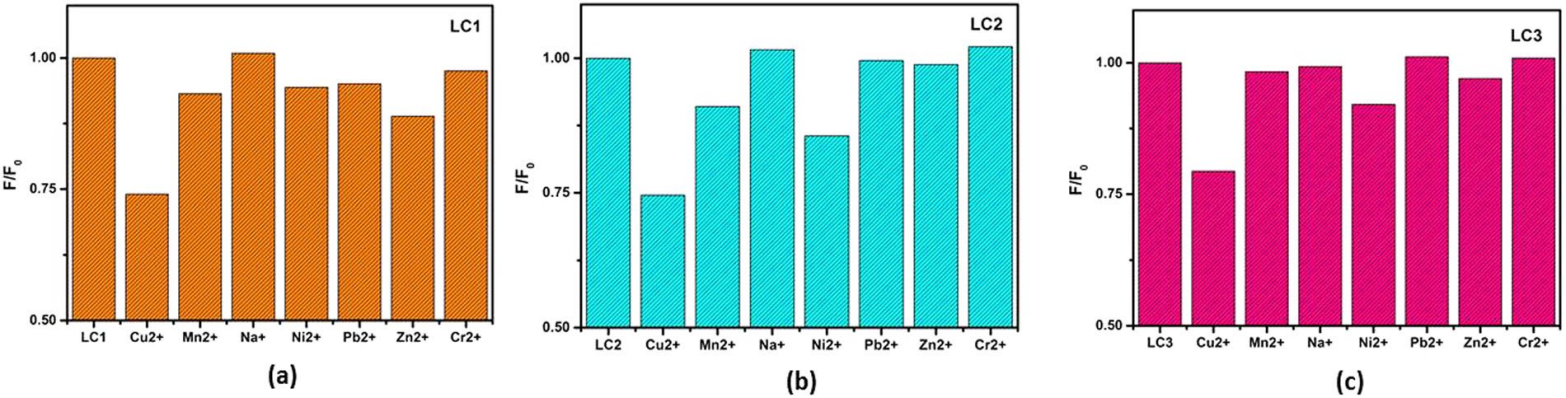
Selectivity of OSDs as probe for metal ions sensing in PBS buffer (pH 7, the concentration of metal ion is 30 ppm, the content of nanoparticles is 2.2 μg mL^−1^).

The lignite derived OSDs have high fluorescent stability and remarkable fluorescence properties; one promising application is as a probe for the metal ion detection in the checking of water contamination and biosensing. It is a green and cost-effective method for metal ion sensing. In the reported study, the potential of the synthesized OSDs from lignite as a low-cost nanocarbon sensor for the metallic ions detection has been reported. The change in fluorescence emission of the OSDs in the buffer in the presence of metal ions is investigated. The metal ions tested include Cu^2+^, Mn^2+^, Na^+^, Ni^2+^, Pb^2+^, Zn^2+^ and Cr^2+^. The results are presented in Fig. 7(a–c), where F_0_ and F are the fluorescence intensities of the probes (at an excitation of 440 nm for LC1 and LC2 and 320 nm for LC3) in the absence and the presence of metal ions. It is interesting to note that out of the seven metal ions tested, Cu^2+^ has the highest selectivity towards fluorescence quenching of all the three OSDs. Chao Hu *et al*.^33^ in their studies ascribed this to the high thermodynamic affinity of Cu^2+^ ions for the NO^-^ chelate groups on the surface of OSDs and the metal-ligand binding kinetics^33–35^. The Cu^2+^ ions quench the fluorescence of the nanodots via electron or energy transfer. Cu^2+^ as a trace element is vital to the human body and is found in natural water. This ion in excess is toxic and can cause kidney and liver malfunctioning. It also causes Parkinson’s and Alzheimer’s diseases. Monitoring the concentration of Cu^2+^ is essential to understand their contribution to health and diseases^36^.

To evaluate the potential of the synthesized nanodots in detecting Cu^2+^ ions in aqueous solution, the fluorescence quenching with different concentration of copper ion is carried out and is presented in Fig. S7(a–d) (Supplementary Information). It is seen that the value of F/F_0_ decreases as the concentration of Cu^2+^ ions changes from 1 ppm to 30 ppm. All three samples show a systematic quenching with a change in Cu^2+^concentration. In comparison, the LC3 sample shows maximum quenching for all the studied concentrations and is found to be a better fluorescent label for the Cu^2+^ detection which can detect to a very low concentration of 125 ppb of metal ions at neutral pH. The Stern-Volmer plot for the nanodots is presented in Fig. S8(a–c) (Supplementary information).

**Figure 8:**
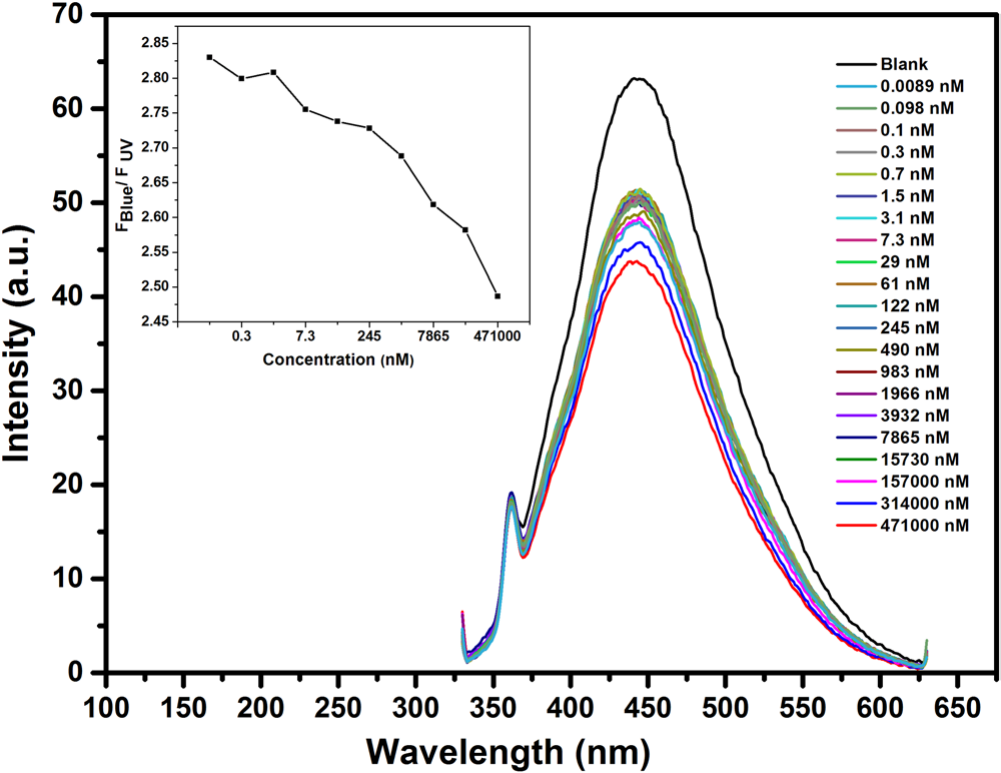
Fluorescent Quenching of Cu^2+^ ion in LC3 at pH 4 (Inset shows the plot of F _blue_/F_UV_ as function of Cu^2+^ concentration).

The copper ion selective quenching of LC3 nanodots are investigated further by varying the pH of the solute (acidic to alkaline) to understand the dependence of pH in quenching of copper ions. The results are depicted in Figs 8 and S9(a,b) (Supplementary information).

It is observed that the fluorescent quenching of the nanoparticles due to Cu^2+^ is dominant in acidic when compared to neutral and basic pH conditions. In the acidic pH, the sample exhibit high sensitivity and could detect as low as 0.0089 nM concentration of the copper ion. Under acidic conditions, the free zigzag sites of the OSDs are protonated, forming a reversible complex between the zigzag sites and H^+^. This leads to the breaking of emissive carbene state and inactivates PL^33–36^. This further lowers the detection limit of Cu^2+^ ions. In alkaline pH, the quenching limit of copper ion is found to be less than 490 nM.

It is also noticed that when sample LC3 is excited at 320 nm, there are two emission peaks appeared one at 360 nm (UV range) and other at 450 nm (Blue range). The intensity of blue peak is continuously changing with the change in concentration of metal ions, while that of 360 nm peak is more or less constant. This property of the OSDs in LC3 is making them a promising candidate for a highly sensitive ratiometric sensor for fluorescent sensing of Cu^2+^ ions^37^. The variation of the fluorescent intensity in the blue region to violet region (F_blue_/F_UV_) as a function of Cu^2+^ concentration is depicted in Fig. 8 (Presented as Inset). It is noticed that they exhibit good sensing from micromolar to the nanomolar concentration of copper ions. The ratiometric sensing can be very useful tool for fluorescence visual detection. This is the first instance of the report of ratiometric sensing of lignite based carbon nanoparticles. Further study on ratiometric sensing and the effect of fluorescence quenching in other pH conditions is necessary to find out the optimum pH for metal detection in real life applications.

In summary, a facile synthesis of highly stable water-soluble organic semiconducting dots from low-grade lignite is reported. The synthesized dots have tunable fluorescent emission from the blue region to green region which arises due to the combination of defect state emission originated from oxygen functional groups, non-uniform particle size and quantum size effect. It is worthwhile to note that all the fractions of synthesized nanocarbon from lignite precursor are exhibiting excitation dependent fluorescence property. They showed pH-dependent luminescence in aqueous solutions without any surface passivation agent. These organic semiconducting dots could be utilized as a label for the detection of Cu^2+^ ions in water with a detection limit as low as 0.0089 nM exhibiting great potential of nanocarbon in sensing application. The OSDs exhibit ratiometric sensing without the addition of any inorganic nanoparticle or modification of the structure.

## Materials and Methods

The precursor used in the present study is Indian Lignite collected from Neyveli lignite mine, Tamil Nadu, India. The as-received sample was powdered before the experiment. Analytically pure chemicals and doubly distilled water were used throughout the experiment.

### Synthesis of nanocarbon structure from lignite

About 5 g of the powdered sample was oxidized using 100 mL dilute HNO_3_ (Water:HNO_3_ :: 5:1) followed by repeated washing with deionized water. Further, it was sonicated for 1 hour followed by magnetic stirring at a temperature of 100 °C (1 hour). The obtained sample was reduced to a pH of 7.7 using NaOH (4 g NaOH in 100 mL H_2_O). This was followed by magnetic stirring without heating for 1 hour. Further, the sample was centrifuged at 12,000 rpm for 30 minutes. After centrifugation, the sample was separated out as residue (LC1-yield of 1.3 g) and supernatant. The supernatant was filtered through a dialysis membrane retaining the large particles inside the membrane (LC2-yield of 0.9 g). The remaining permeate which are the smaller particles (LC3-yield of 1.7 g) were collected outside the membrane separately. All the three samples (LC1, LC2 and LC3) were dried by slow evaporation at 80 °C. The combined production yield of the nanostructure is found to be 78% which is very high production yield for gram scale production of fluorescent carbon dots.

### Characterization

Raman measurements were recorded at a wavelength of 514.5 nm using confocal Raman microscope by Renishaw (In Via Raman). TEM analyses of the samples were done using a JEOL JEM-2100 model. The fluorescent study was carried out with an RF-5301 PC, Shimadzu fluorescence spectrometer. Functional groups were qualitatively identified by a Fourier transform infrared spectrometer (Thermo Nicolet 370 spectrophotometer). Atomic force microscope (Witec Alpha 300RA) was used for the height profile analysis. The UV/Vis spectrometer (Ocean optics JAZ series) was used for acquiring absorption spectra. The CHNS analysis was carried out using Elementar Vario EL111 elemental analyzer.

### Detection of metal ion

The utility of the synthesized nanocarbon as a fluorescent sensor for detection of metal ions was performed at room temperature in PBS (pH 7.0) buffer. Cu^2+^, Mn^2+^, Na^+^, Ni^2+^, Pb^2+^, Zn^2+^ and Cr^2+^ solutions were prepared with a concentration of 30 ppm. 100 μL of each metal ion solution was added separately to 1000 μL PBS buffer. Three samples LC1, LC2 and LC3 (2.2 μg mL^−1^) were added separately to each buffer- ion solution. The fluorescence emission spectrum was noted after shaking the mixture for 5 seconds. The copper ion selective quenching of LC3 at different pH conditions (acidic to alkaline) was investigated.

## Electronic supplementary material

Supplementary Information
