# Supplementary material for: Tunable direct band gap photoluminescent organic semiconducting nanoparticles from lignite
Source: Sci Rep. 2017 Dec 21;7:18012. doi: 10.1038/s41598-017-18338-2 (PMC5740083; doi:10.1038/s41598-017-18338-2)
Supplement: Supplementary file 1 — Supplementary Information [file 41598_2017_18338_MOESM1_ESM.pdf]

Supplementary Information

Tunable direct band gap photo luminescent organic semiconducting nanoparticles from Lignite

Manoj.B, Ashlin M Raj, George Thomas Chirayil

Department of Physics, Christ University, Bengaluru, Karnataka, India, 560029

E-mail: manoj.b@christuniversity.in, ashlinmrj@gmail.com

Table. S1: CHNS Analysis of LC1, LC2 and LC3

Sample	C%	H%	N%	S%	O%	O/C ratio	N/C ratio
LC1	37.79	3.69	4.65	0.93	52.94	1.40	0.12
LC2	34.83	2.51	2.41	1.32	58.93	1.69	0.07
LC3	4.30	0.66	13.51	0.16	81.37	18.92	3.14

Table. S 2: Fluorescent life time of the synthesized oxygenated semiconductor dots

Sample	τ_1 (ns)	τ_2 (ns)	τ_3 (ns)	B_1	B_2	B_3	τ_{avg} (ns)
LC1	3.548432	0.8002666	10.28164	48.37	17.79	33.84	5.338
LC2	3.084514	0.8002666	10.28164	45.15	18.82	36.03	5.231
LC3	3.651468	0.8046068	10.14289	46.83	17.10	36.07	5.506

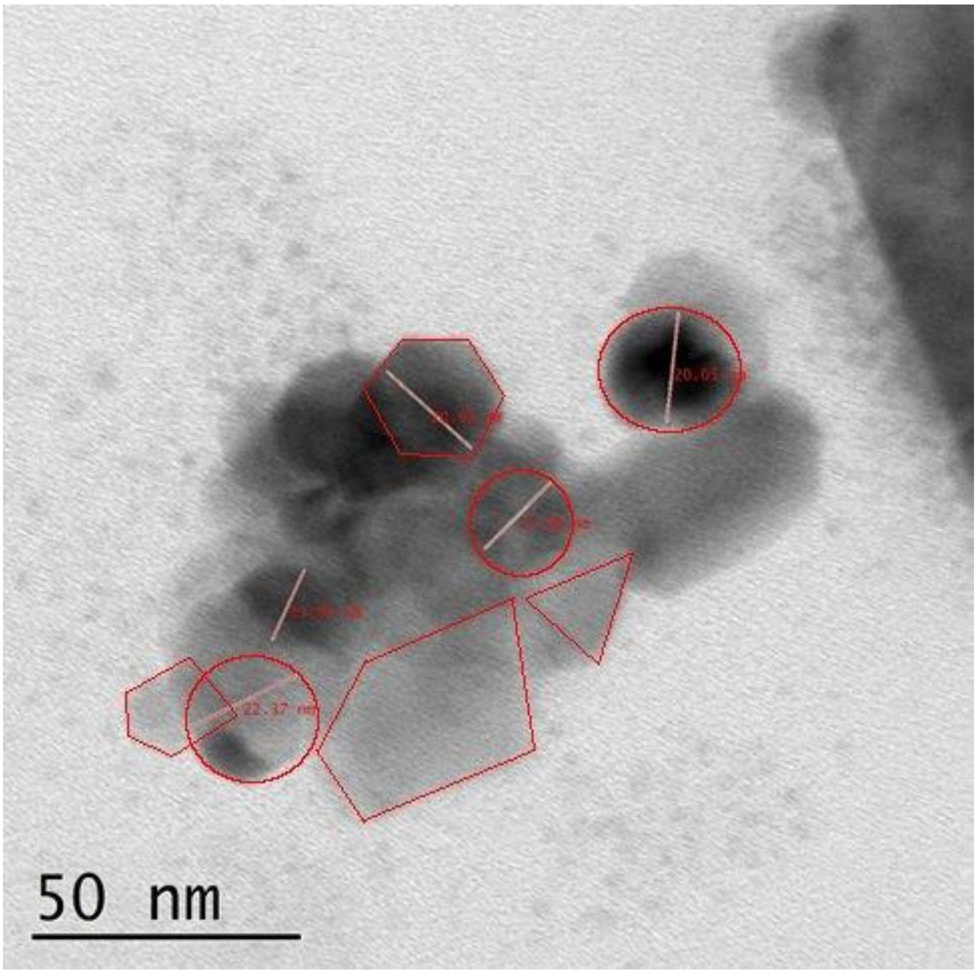

Figure S 1: TEM image of LC1 showing particle size of ~10 nm to 23 (at a magnification of 50 nm). There are hexagonal, pentagonal, trigonal and spherical nanocarbon dots observed.

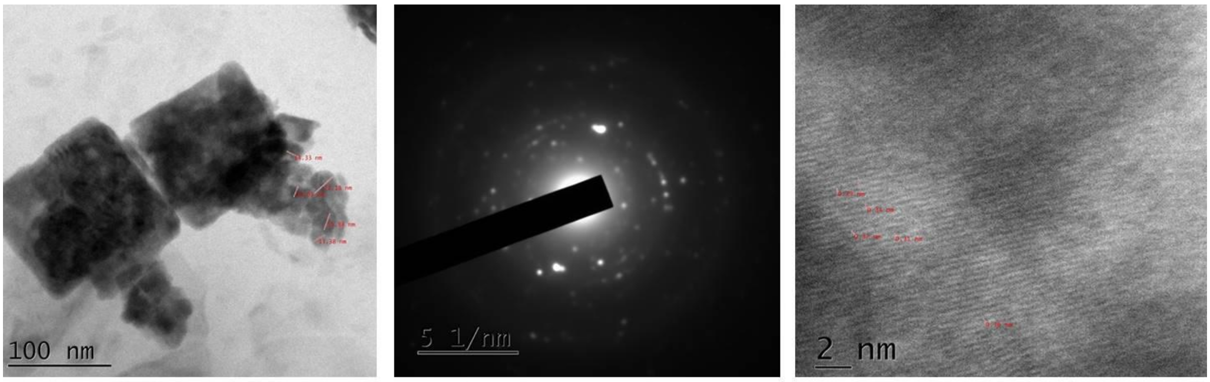

(a) (b) (c)

Figure S 2 (a-c): (a) TEM image of LC2 showing particle size of ~10 nm to 17 nm (at a magnification of 100 nm). The dots are stacked together to form cubical structure. (b) SAED

pattern of the highlighted area (c) Lattice fringe pattern of the carbon nanoparticles is also noticed.

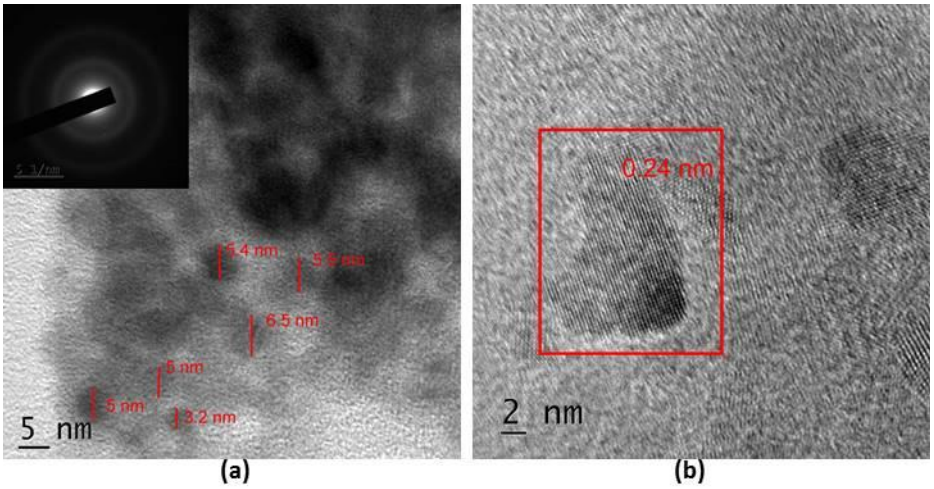

Figure S 3 (a, b): a) TEM image of LC3 showing nanodots of ~ 5 nm size (SAED pattern is shown in inset) b) TEM image showing crystalline nature of LC3 (Lattice fringe ~ 0.24 nm). The dots are mixture of amorphous and nano crystalline structure

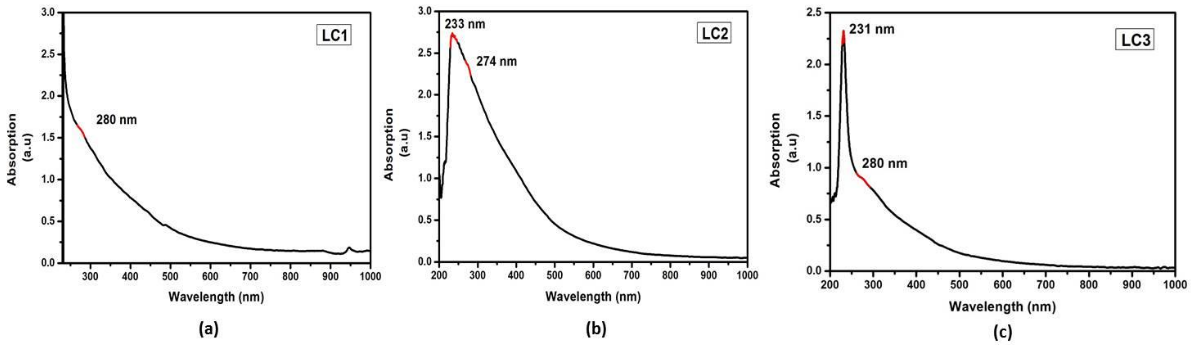

Figure S 4 (a-c): UV Visible spectrum of LC1, LC2 and LC3

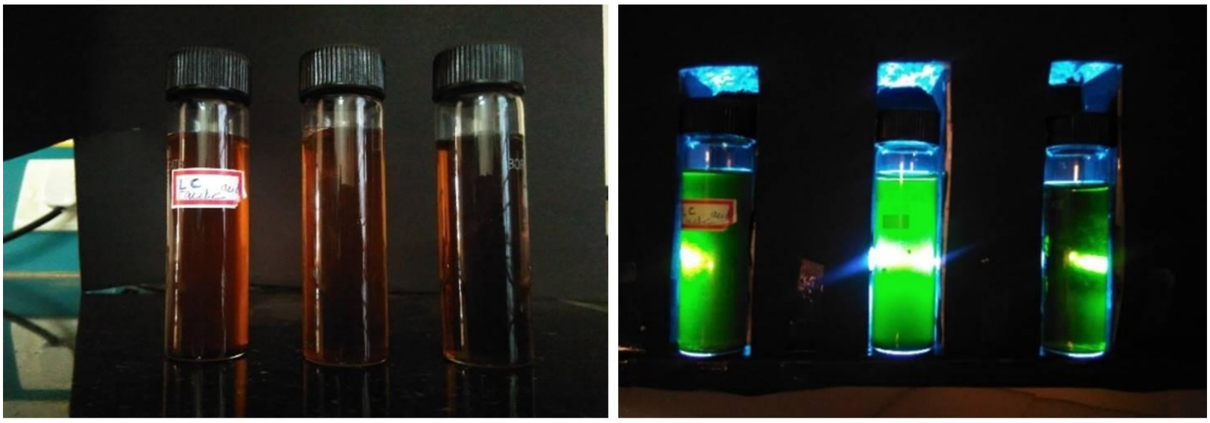

(a) (b)

Figure S 5 (a, b): a) Without UV exposure b) Under UV exposure (365 nm)

The product is highly stable and shows dark green fluorescence even after 24 months.

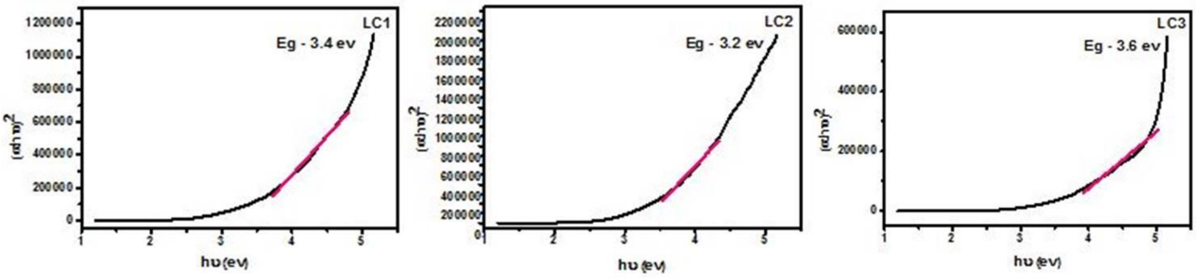

Figure S 6 (a-c): Tauc Plot of LC1, LC2 and LC3 with energy gap

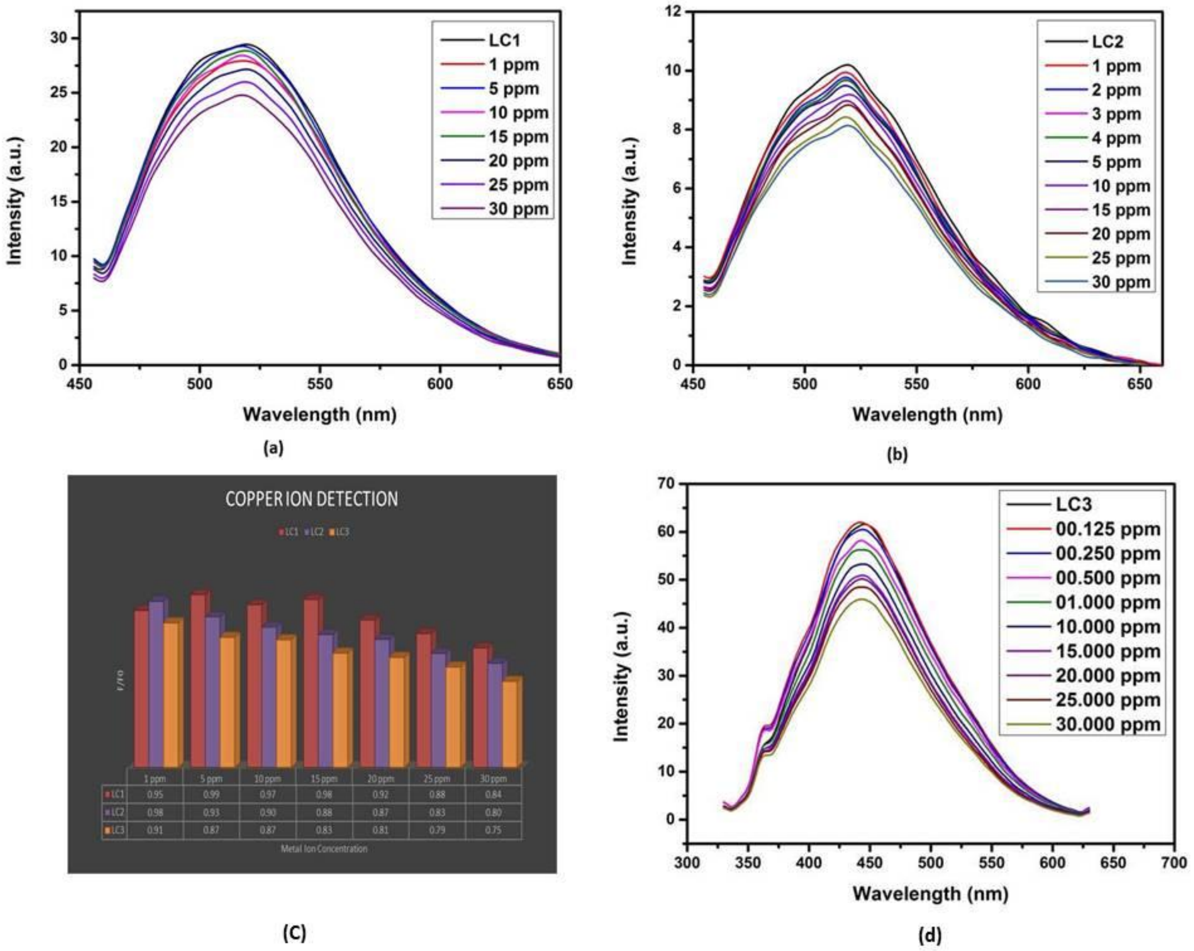

Figure S 7 (a - d): Fluorescence response of OSDs dispersion upon addition of various concentration of Cu^{2+} (in aqueous solution)

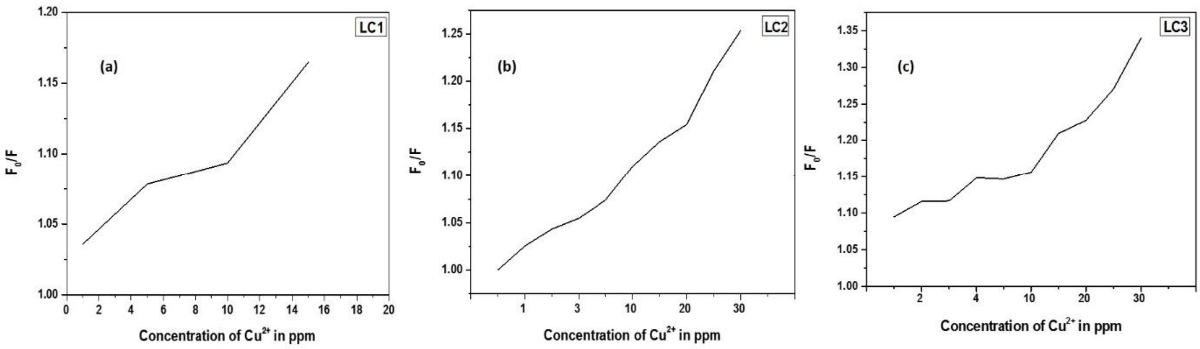

Figure S 8 (a-c): Stern Volmer plot of LC1, LC2 and LC3

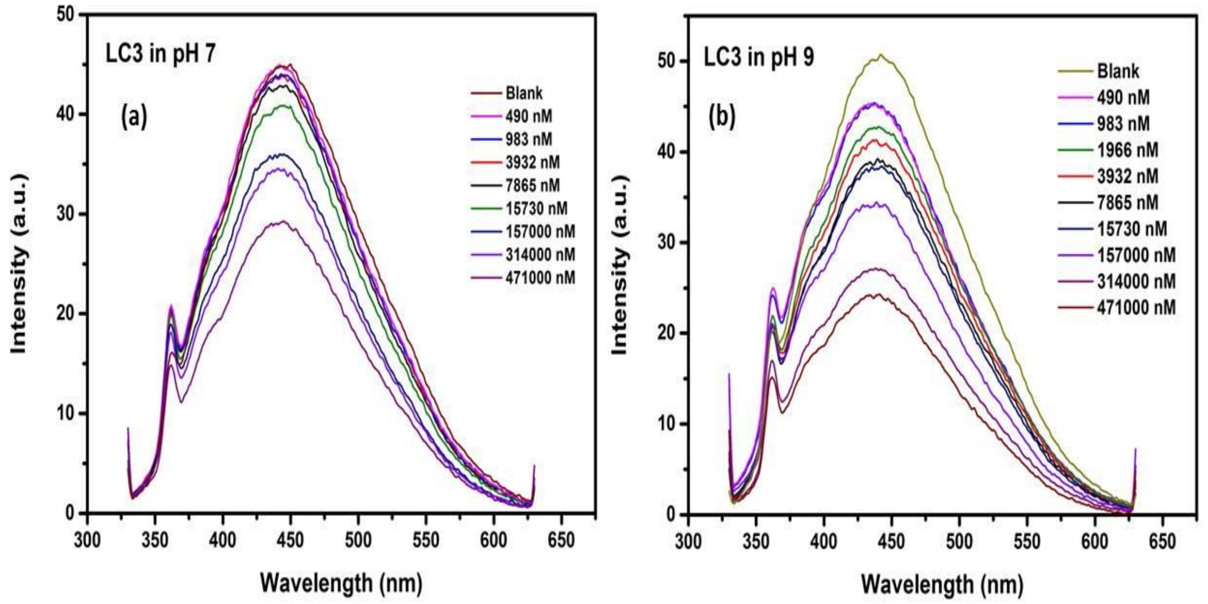

Figure S 9 (a, b): Fluorescent Quenching of Cu^{2+} ion in OSDs (a) At pH 7 (b) At pH 9 (fluorescent quenching in the range 471000nM to 490 nM is shown)
